# Supplementary figures and images for: Connexin 43 hemichannels regulate mitochondrial ATP generation, mobilization, and mitochondrial homeostasis against oxidative stress
Source: eLife. 2022 Nov 8;11:e82206. doi: 10.7554/eLife.82206 (PMC9642995; doi:10.7554/eLife.82206)

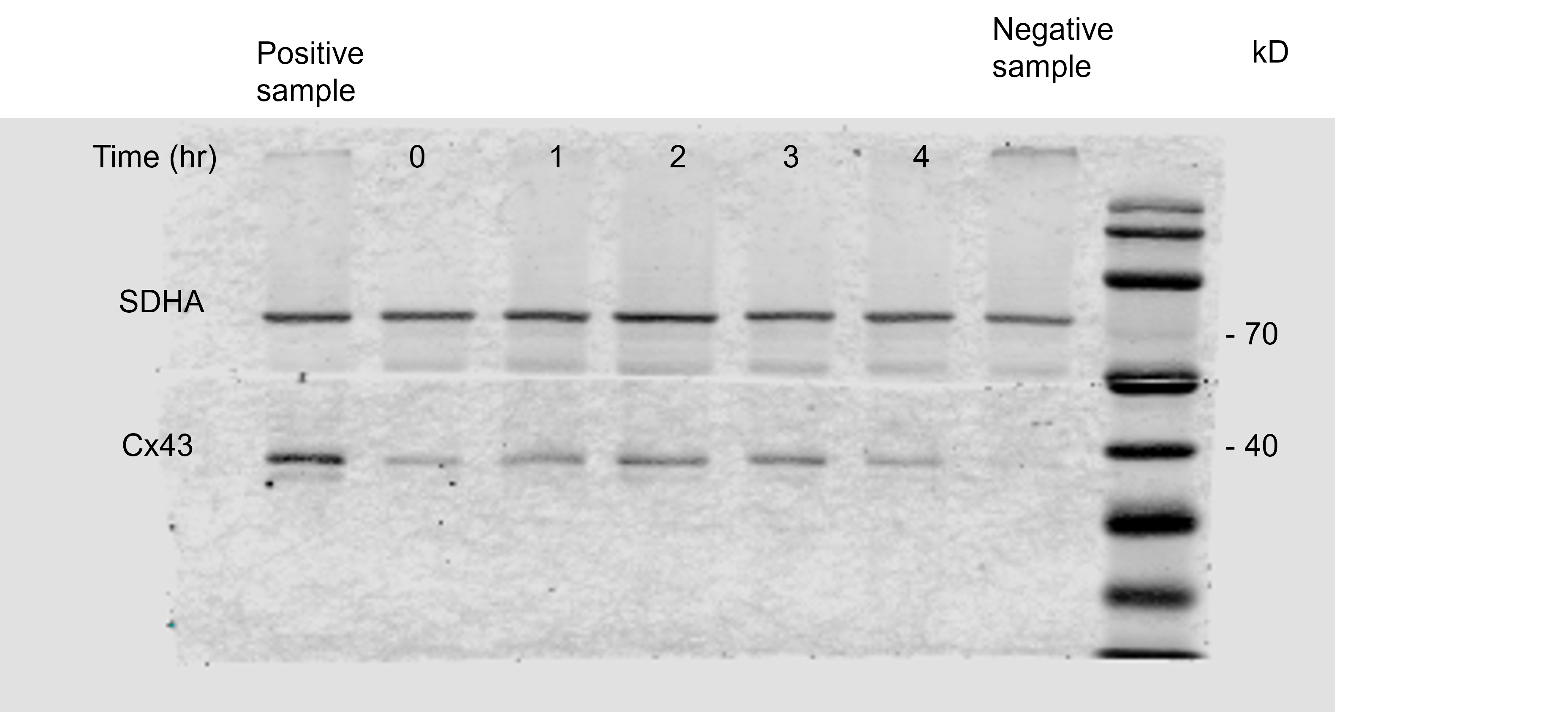

Supplement: Figure 1—source data 2. [file elife-82206-fig1-data2.zip › Figure 1-Source data 2.tif]

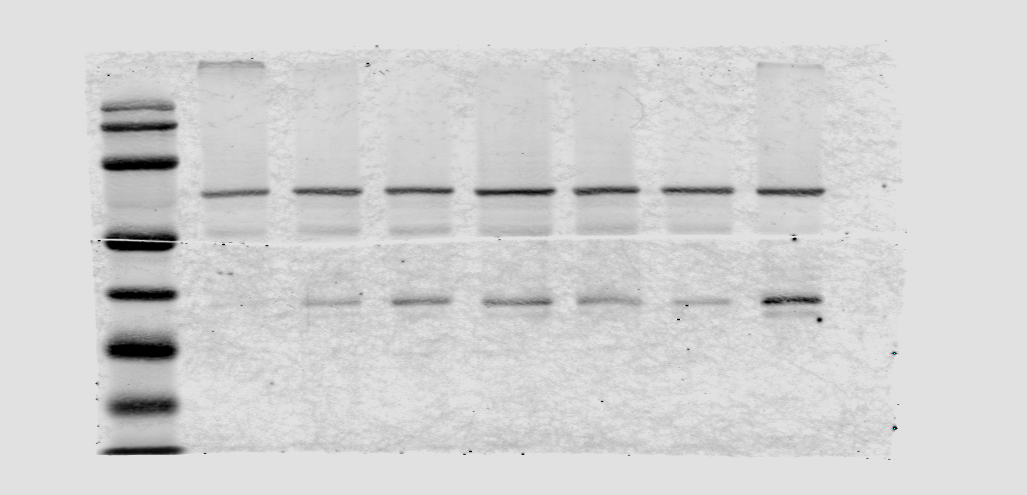

Supplement: Figure 1—source data 2. [file elife-82206-fig1-data2.zip › gel 2.tif]

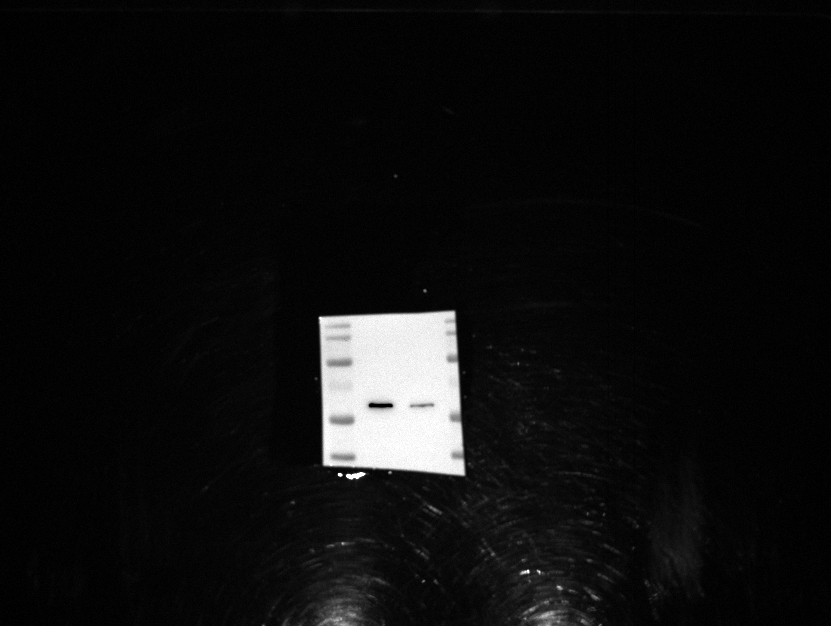

Supplement: Figure 1—figure supplement 1—source data 1. [file elife-82206-fig1-figsupp1-data1.zip › PDI_1.png]

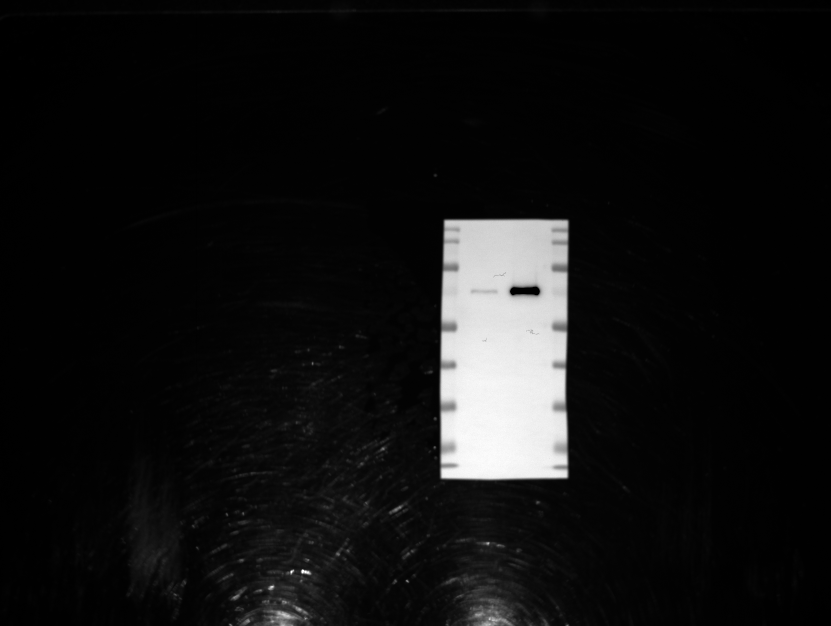

Supplement: Figure 1—figure supplement 1—source data 1. [file elife-82206-fig1-figsupp1-data1.zip › SDHA_0.png]

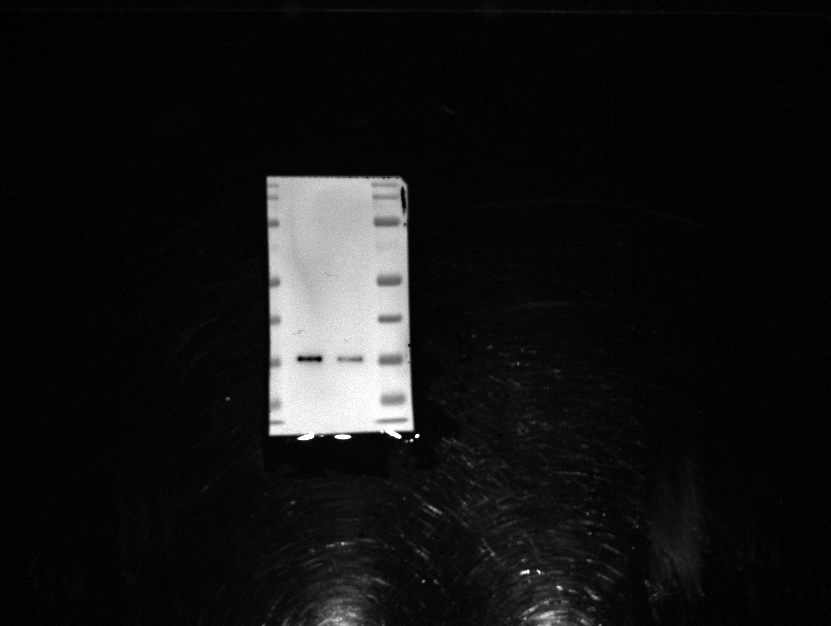

Supplement: Figure 1—figure supplement 1—source data 1. [file elife-82206-fig1-figsupp1-data1.zip › STX6_1.png]

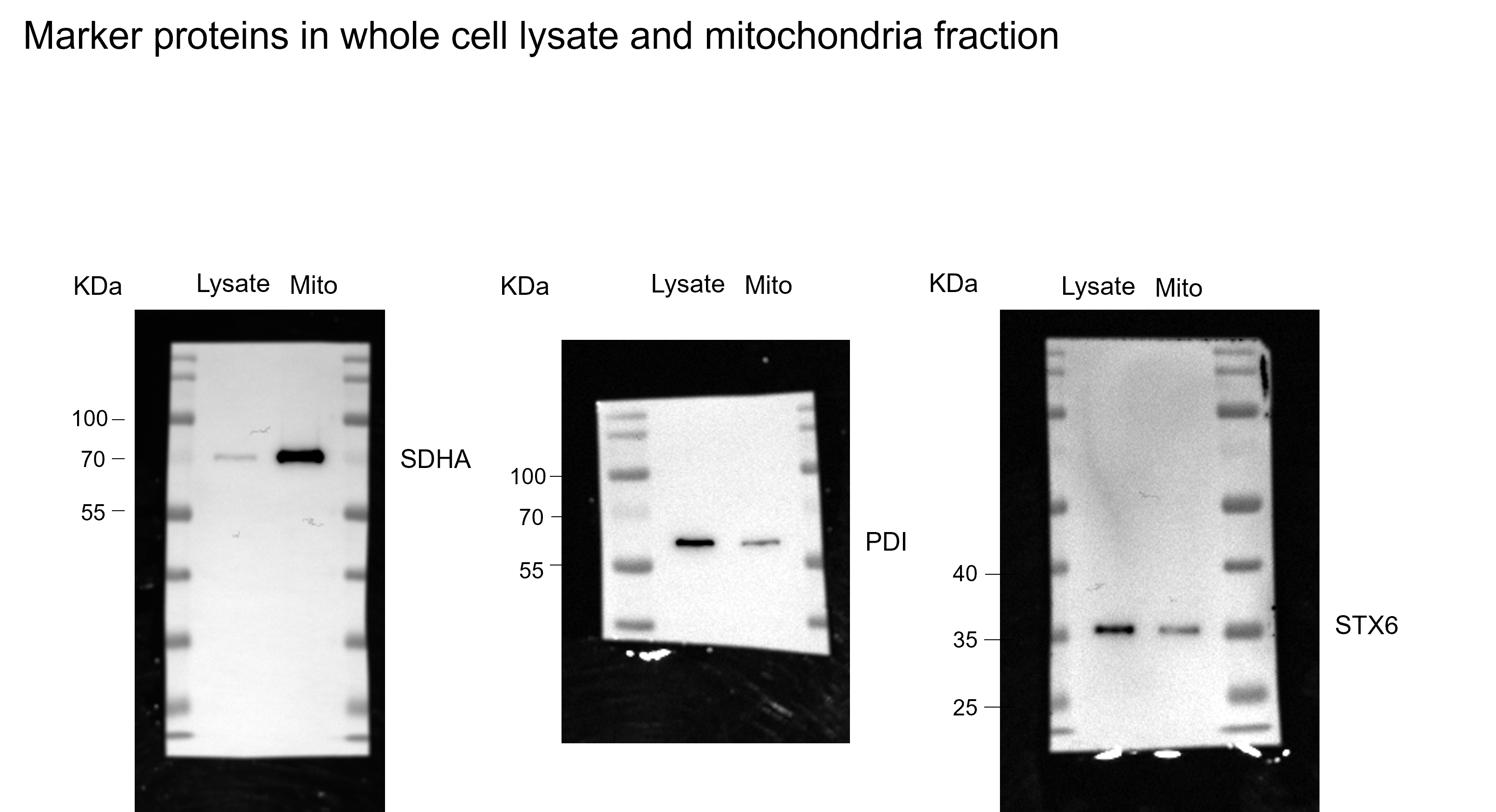

Supplement: Figure 1—figure supplement 1—source data 1. [file elife-82206-fig1-figsupp1-data1.zip › Figure 1- supplement 1-Source Data 1.tif]

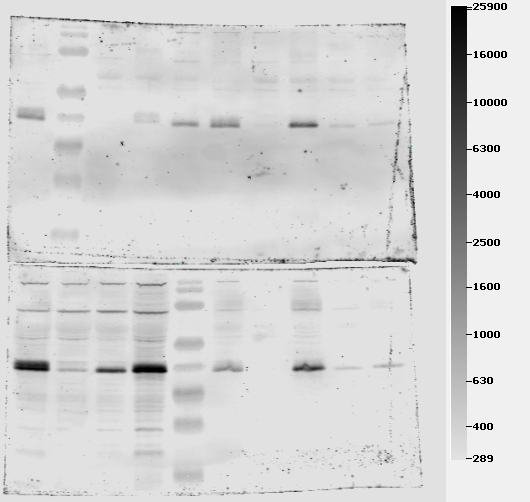

Supplement: Figure 2—source data 1. [file elife-82206-fig2-data1.zip › CT.tif]

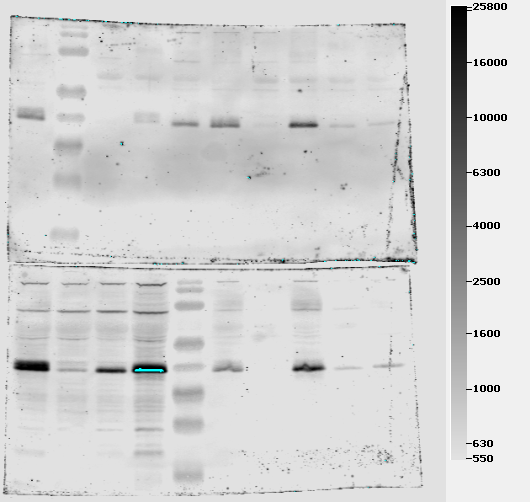

Supplement: Figure 2—source data 1. [file elife-82206-fig2-data1.zip › CT-2.tif]

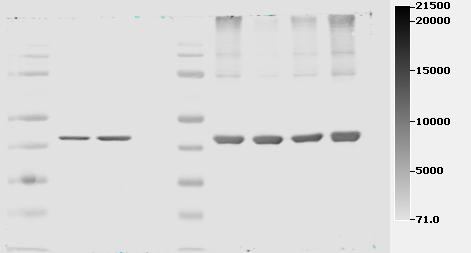

Supplement: Figure 2—source data 1. [file elife-82206-fig2-data1.zip › Cx43 KO Actb-1.png]

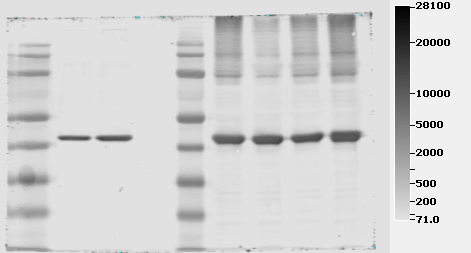

Supplement: Figure 2—source data 1. [file elife-82206-fig2-data1.zip › Cx43 KO actb-2.png]

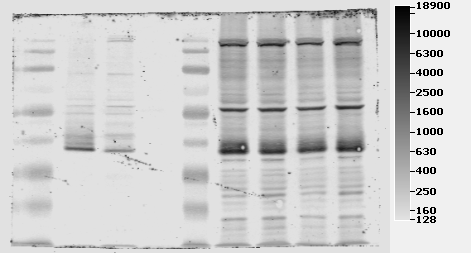

Supplement: Figure 2—source data 1. [file elife-82206-fig2-data1.zip › Cx43 KO CT Ab-2.png]

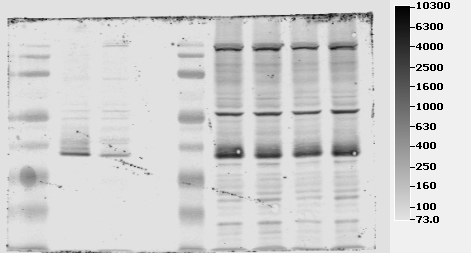

Supplement: Figure 2—source data 1. [file elife-82206-fig2-data1.zip › Cx43 KO CT Ab-3.png]

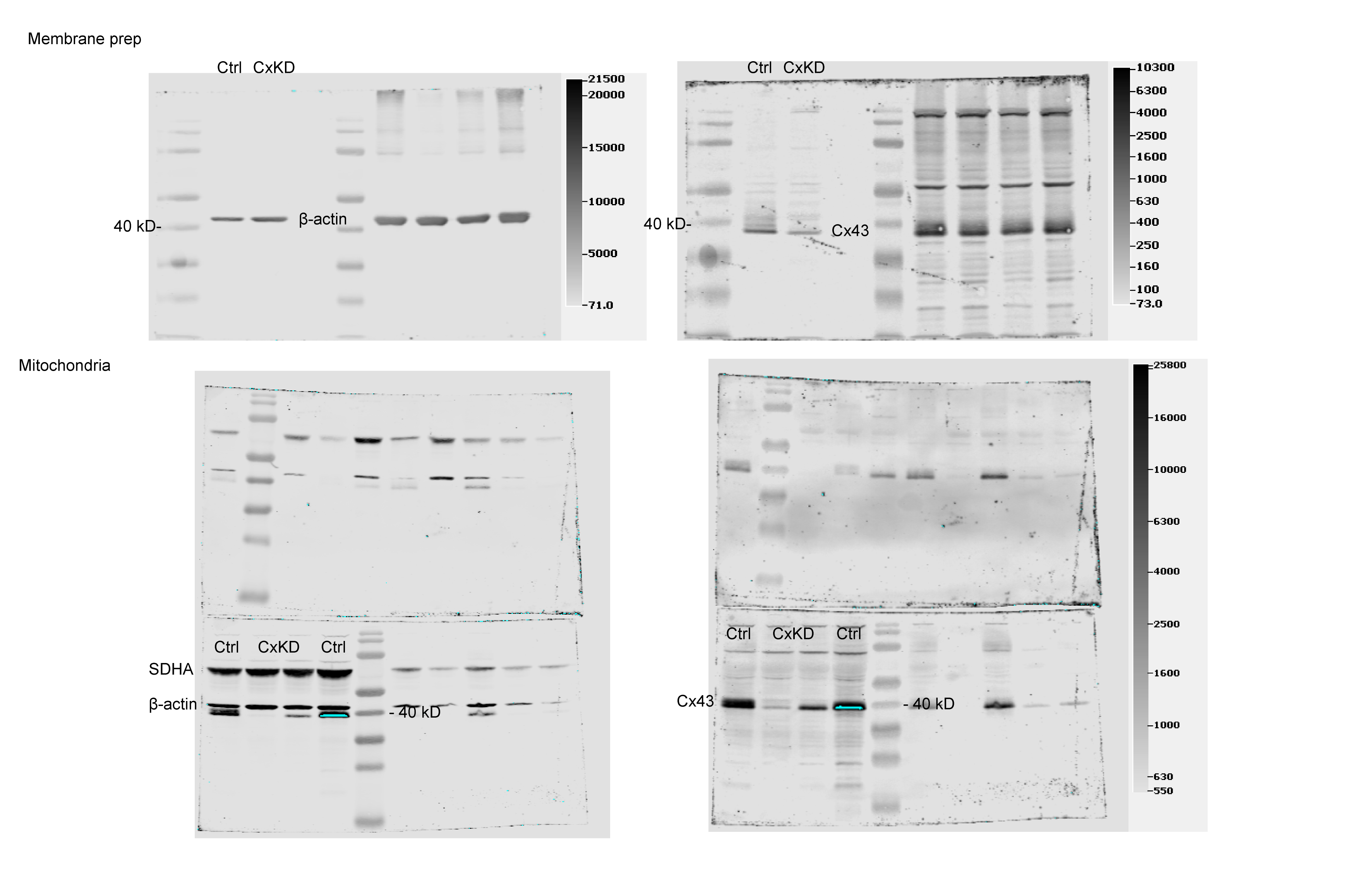

Supplement: Figure 2—source data 1. [file elife-82206-fig2-data1.zip › Figure 2-source data 1.tif]

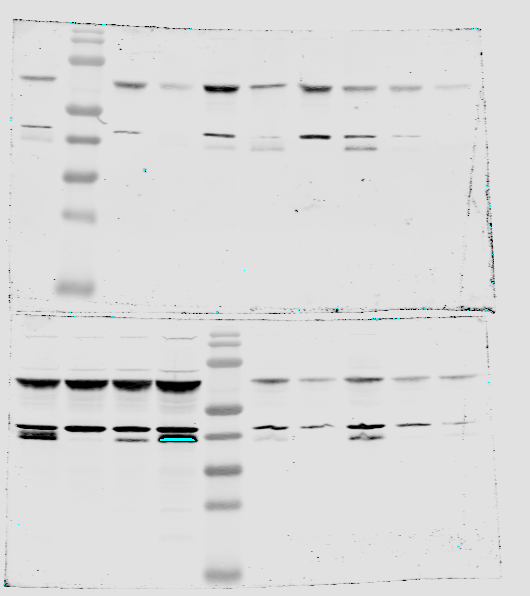

Supplement: Figure 2—source data 1. [file elife-82206-fig2-data1.zip › SDHA actb CT-2.tif]

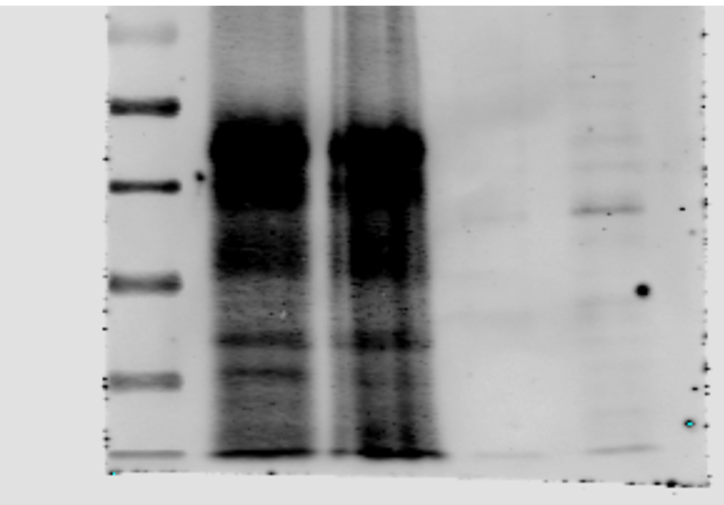

Supplement: Figure 7—source data 1. [file elife-82206-fig7-data1.zip › Cx43-1.png]

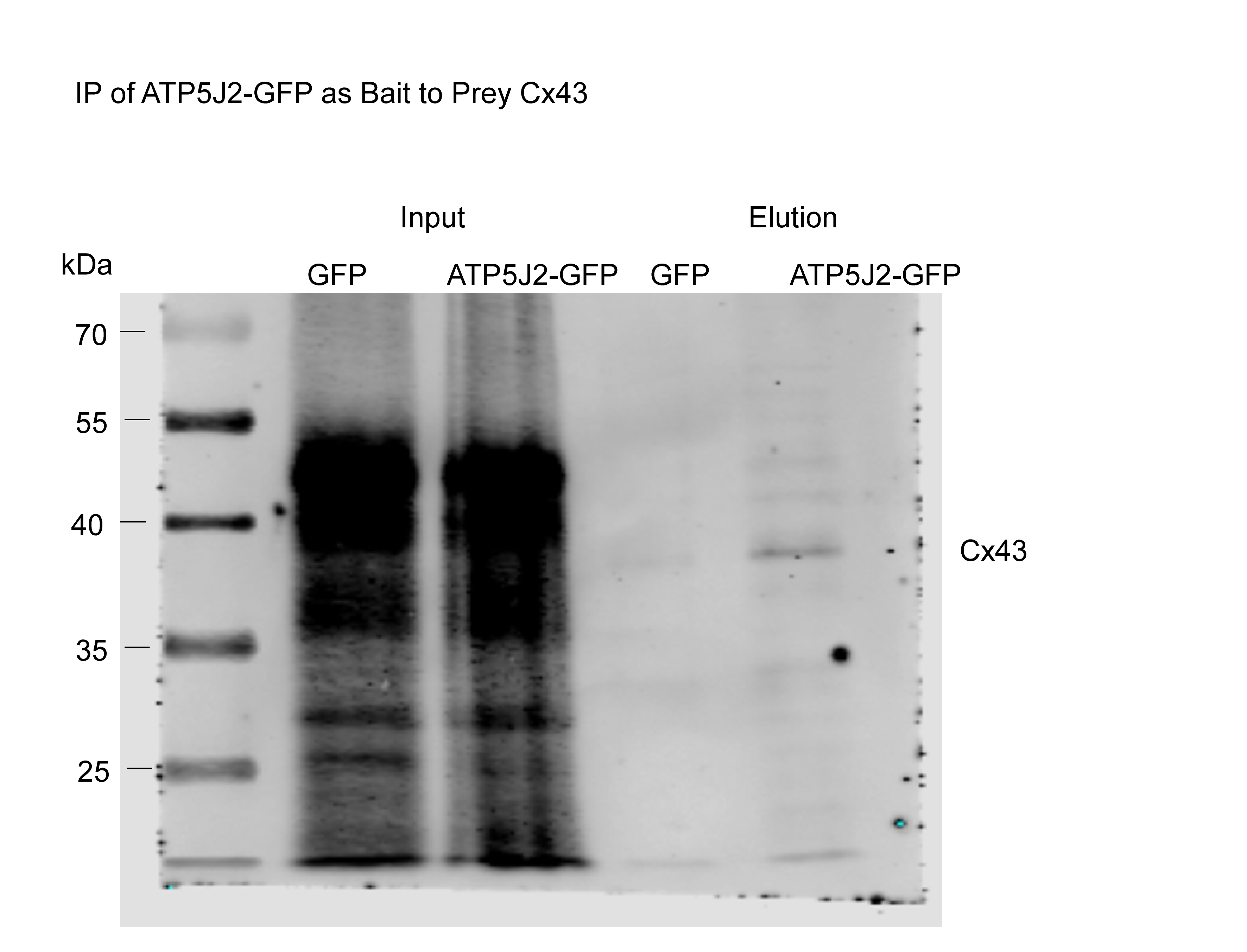

Supplement: Figure 7—source data 1. [file elife-82206-fig7-data1.zip › Figure 7-Source data 1.tif]

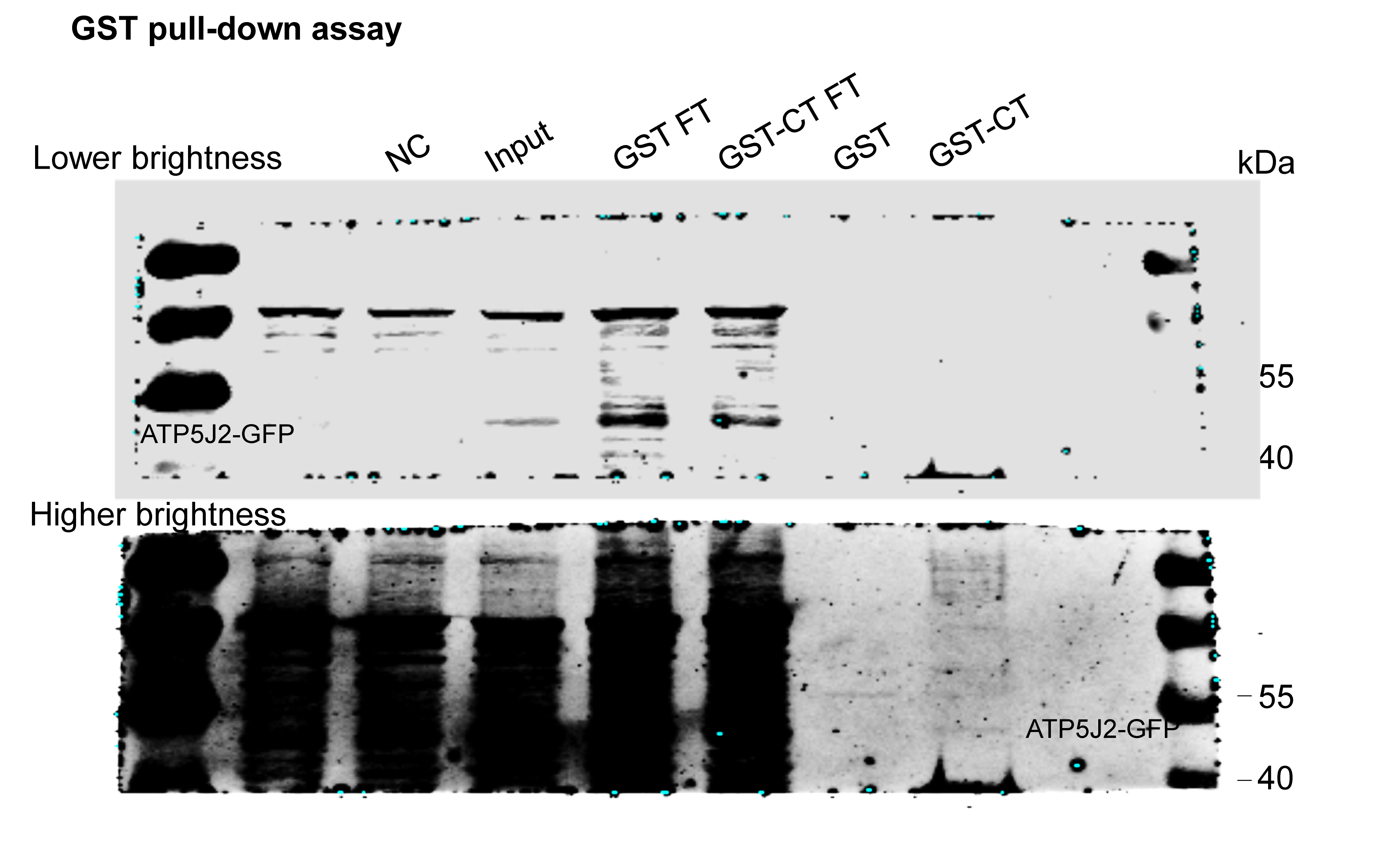

Supplement: Figure 7—source data 2. [file elife-82206-fig7-data2.zip › Figure 7-Source data 2.tif]

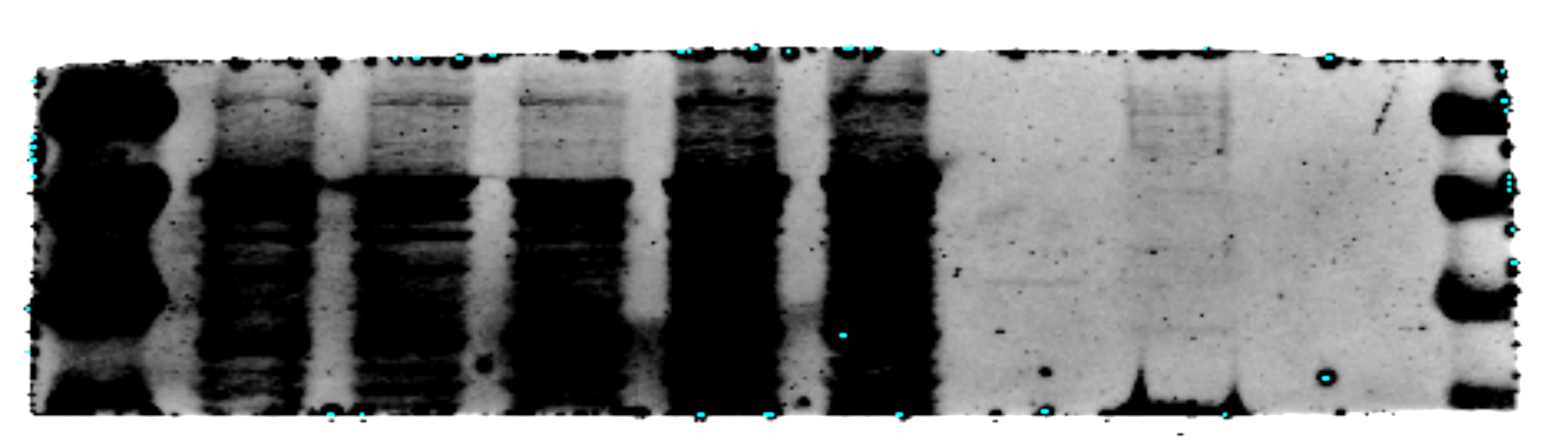

Supplement: Figure 7—source data 2. [file elife-82206-fig7-data2.zip › GFP-1.png]

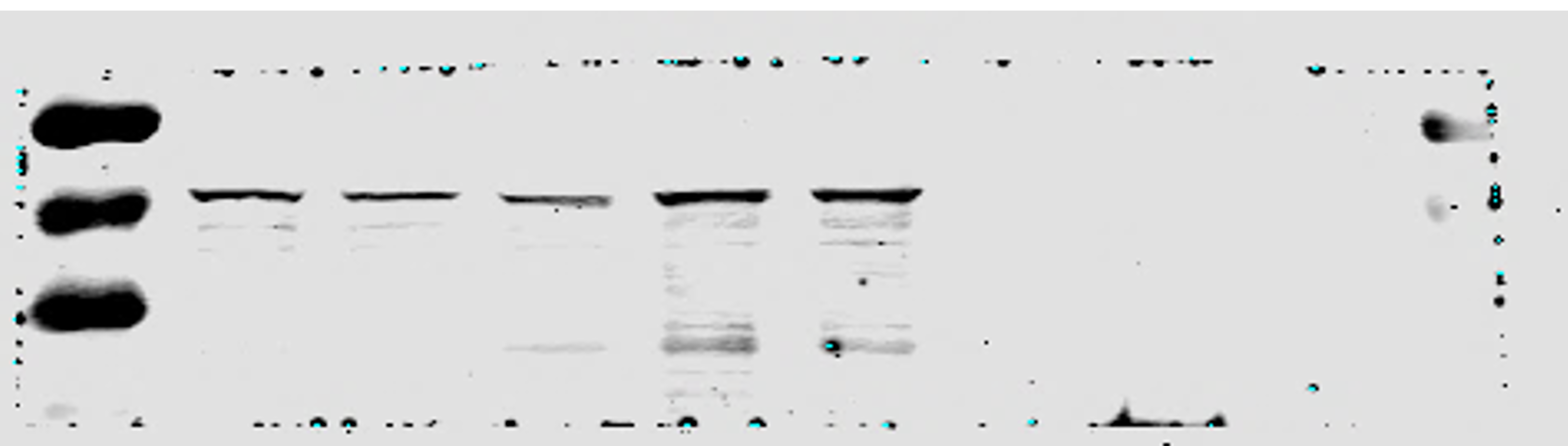

Supplement: Figure 7—source data 2. [file elife-82206-fig7-data2.zip › GFP-2.png]

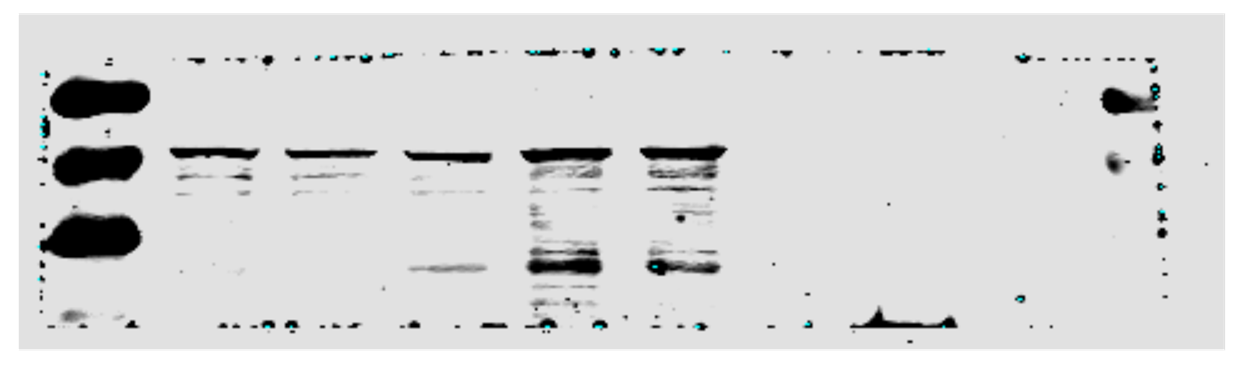

Supplement: Figure 7—source data 2. [file elife-82206-fig7-data2.zip › GFP-3.png]

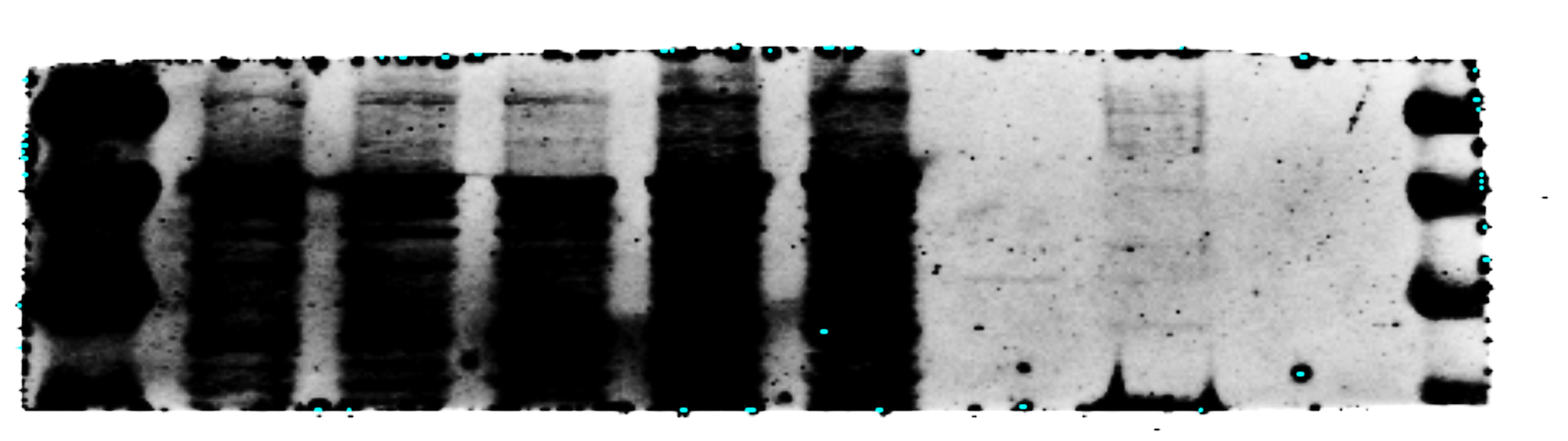

Supplement: Figure 7—source data 2. [file elife-82206-fig7-data2.zip › GFP-4.png]

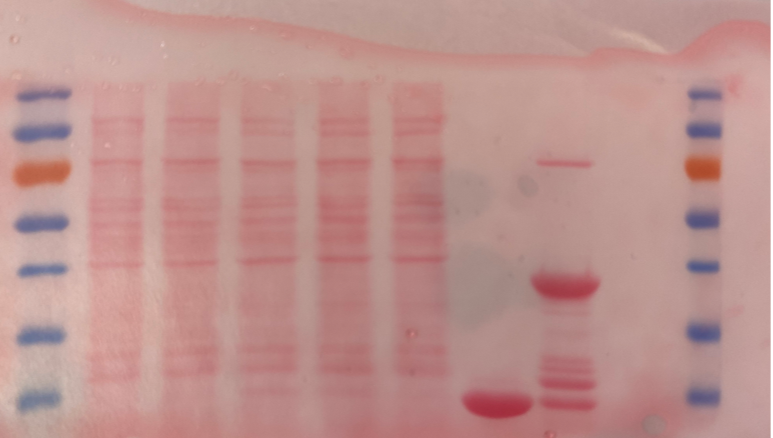

Supplement: Figure 7—source data 2. [file elife-82206-fig7-data2.zip › ponceau s-2.png]
